# Bis(2,4,6-triamino-1,3,5-triazin-1-ium) pyrazine-2,3-dicarboxyl­ate tetra­hydrate: a synchrotron radiation study

**DOI:** 10.1107/S1600536810017290

**Published:** 2010-05-15

**Authors:** Hossein Eshtiagh-Hosseini, Azam Hassanpoor, Laura Canadillas-Delgado, Masoud Mirzaei

**Affiliations:** a Department of Chemistry, School of Sciences, Ferdowsi University of Mashhad, Mashhad, Iran; b Instituto de Ciencia de Materiales de Aragón, CSIC-Universidad de Zaragoza, C/ Pedro Cerbuna 12, E-50009 Zaragoza, Spain

## Abstract

The title compound, 2C_3_H_7_N_6_^+^·C_6_H_2_N_2_O_4_^2−^·4H_2_O or (tataH)_2_(pzdc)·4H_2_O, was synthesised by a reaction between pyrazine-2,3-dicarboxylic acid (H_2_pzdc) as a proton donor and 2,4,6-triamino-1,3,5-triazin (tata) as a proton acceptor. In the crystal structure, an extensive series of O—H⋯O, O—H⋯N, N—H⋯O and N—H⋯N hydrogen bonds generates a three-dimensional framework with the hydrogen bonding involving most donor and acceptor centers. π–π stacking inter­actions are also observed between adjacent triazine rings, with centroid–centroid distances of 3.4994 (8) and 3.5922 (7) Å.

## Related literature

For related structures, see Xu *et al.* (1999 ▶); Wang *et al.* (2008 ▶); Liu *et al.* (2008 ▶); Moghimi *et al.* (2007 ▶); Smith *et al.* (2006*a* ▶,*b* ▶); Zafar *et al.* (2000 ▶).
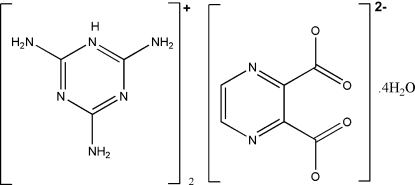


## Experimental

### 

#### Crystal data

2C_3_H_7_N_6_^+^·C_6_H_2_N_2_O_4_^2−^·4H_2_O
*M* *_r_* = 492.45
Triclinic, 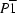

*a* = 7.0200 (14) Å
*b* = 9.763 (2) Å
*c* = 15.397 (3) Å
α = 101.06 (3)°
β = 99.82 (3)°
γ = 96.98 (3)°
*V* = 1007.4 (4) Å^3^
*Z* = 2
Synchrotron radiation
λ = 0.73800 Å
μ = 0.14 mm^−1^
*T* = 100 K
0.15 × 0.04 × 0.03 mm

#### Data collection

Huber single-axis diffractometer
34176 measured reflections
4609 independent reflections
4314 reflections with *I* > 2σ(*I*)
*R*_int_ = 0.086

#### Refinement

*R*[*F* ^2^ > 2σ(*F* ^2^)] = 0.043
*wR*(*F* ^2^) = 0.117
*S* = 1.07
4609 reflections
339 parameters
H atoms treated by a mixture of independent and constrained refinement
Δρ_max_ = 0.36 e Å^−3^
Δρ_min_ = −0.39 e Å^−3^

### 

Data collection: *MXCUBE* (Gabadinho & McSweeney, 2010 ▶); cell refinement: *HKL-2000* (Otwinowski & Minor, 1997 ▶); data reduction: *HKL-2000*; program(s) used to solve structure: *SHELXS97* (Sheldrick, 2008 ▶); program(s) used to refine structure: *SHELXL97* (Sheldrick, 2008 ▶); molecular graphics: *ORTEP-3 for Windows* (Farrugia, 1997 ▶); software used to prepare material for publication: *WinGX* (Farrugia, 1999 ▶).

## Supplementary Material

Crystal structure: contains datablocks global, I. DOI: 10.1107/S1600536810017290/sj2786sup1.cif

Structure factors: contains datablocks I. DOI: 10.1107/S1600536810017290/sj2786Isup2.hkl

Additional supplementary materials:  crystallographic information; 3D view; checkCIF report

## Figures and Tables

**Table 1:** Hydrogen-bond geometry (Å, °)

*D*—H⋯*A*	*D*—H	H⋯*A*	*D*⋯*A*	*D*—H⋯*A*
N3—H3*A*⋯O2	0.86	1.98	2.7726 (16)	153
N6—H6*A*⋯N5^i^	0.86	2.10	2.9449 (17)	169
N6—H6*B*⋯O3	0.86	2.19	2.8206 (17)	130
N6—H6*B*⋯O2	0.86	2.32	3.0378 (15)	141
N7—H7*A*⋯O2*W*^ii^	0.86	2.17	3.0080 (16)	164
N7—H7*B*⋯O4*W*	0.86	2.04	2.8407 (19)	156
N8—H8*A*⋯N9^iii^	0.86	2.39	3.2495 (17)	180
N8—H8*B*⋯O3^i^	0.86	2.03	2.8842 (16)	170
N11—H11⋯O1	0.86	1.98	2.7883 (17)	155
N12—H12*A*⋯O4^iv^	0.86	1.92	2.7557 (15)	162
N12—H12*B*⋯O3*W*^iv^	0.86	2.46	2.9981 (18)	121
N12—H12*B*⋯O1	0.86	2.47	3.1643 (17)	139
N13—H13*A*⋯N4^iii^	0.86	2.08	2.9275 (16)	167
N13—H13*B*⋯O2*W*^v^	0.86	2.10	2.8713 (16)	148
N14—H14*B*⋯O1*W*^vi^	0.86	2.21	2.9756 (16)	148
O1*W*—H1*WA*⋯O2	0.89 (3)	1.87 (2)	2.7263 (16)	163 (2)
O1*W*—H1*WB*⋯O1^vii^	0.93 (3)	1.95 (3)	2.8342 (16)	159 (2)
O2*W*—H2*WA*⋯O1*W*^vi^	0.85 (3)	2.07 (3)	2.8623 (16)	154 (3)
O2*W*—H2*WB*⋯O4	0.93 (3)	1.83 (3)	2.7533 (17)	171 (3)
O3*W*—H3*WA*⋯N2	0.99 (3)	1.97 (3)	2.9589 (16)	170 (2)
O3*W*—H3*WB*⋯O1^viii^	0.92 (3)	2.07 (3)	2.9810 (15)	176 (2)
O4*W*—H4*WA*⋯O3*W*^iv^	0.93 (3)	1.88 (3)	2.7846 (17)	164 (2)
O4*W*—H4*WB*⋯N1^ix^	0.89 (3)	2.08 (3)	2.9219 (17)	156 (2)

Symmetry codes: (i) 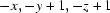; (ii) 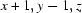; (iii) 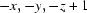; (iv) 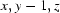; (v) 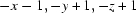; (vi) 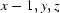; (vii) 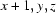; (viii) 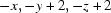; (ix) 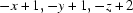.

**Fig. 1.**
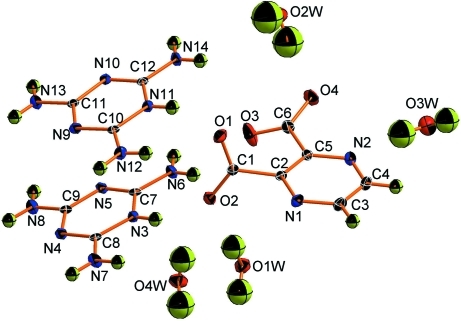
The asymmetric unit of the title compound with ellipsoids drawn at the 80% probability level.

**Fig. 2.**
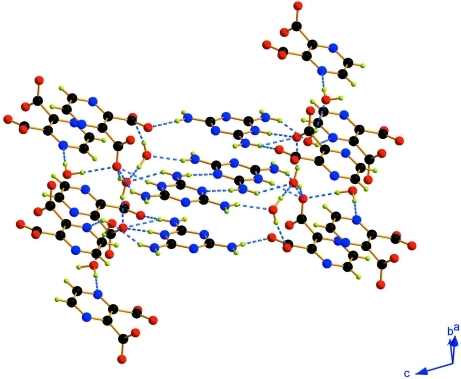
Crystal packing of 1 with hydrogen bonds drawn as dashed lines.

## supplementary crystallographic information

### Comment

In recent years, proton transfer from appropriate H-donors to H-acceptors has emerged as a method for preparing self-assembling systems  (Zafar *et al.*, 2000). In such systems there are a variety of non-covalent interactions such as H-bonding and aromatic π···π stacking (Moghimi *et al.*, 2007). There have been several attempts to prepare proton transfer compounds involving carboxylic acids and amines. For example ion pairs have been reported between H_2_pzdc and various organic bases such as 8-hydroxy quinoline (Smith *et al.*, 2006a) and guanidine (Smith *et al.*, 2006b).  In this work, we report a new proton transfer compound obtained from H_2_pzdc as a proton donor and tata as an acceptor. The molecular structure of the title compound is shown in Fig. 1. The structure of this compound contains two monocationic (tataH)^+^, one (pzdc)^2-^ species and four water molecules.

In the crystal structure, there are several π···π interactions between adjacent triazine rings with centroid···centroid distances of 3.4994 (8) and 3.5922 (7)Å. Extensive H-bonding interactions also occur with H···A distances ranging from 1.86 to 2.47Å. These together with the π-π stacking, connect the different components giving rise the final three-dimensional supramolecular structure (Fig. 2).

### Experimental

By refluxing 0.118 mmol (0.02 g) H_2_pzdc and 0.237 mmol (0.03 g) tata in 15 ml water for 6 h at 70 C°, a colorless solution was obtained. This solution gave colorless needle-like crystals of the title compound after slow evaporation of the solvent at RT.

### Refinement

The H atoms of the ligands were treated as riding with distances 0.86 (N—H), 0.93 (C—H), and U_iso_(H) = 1.2 U_eq_(C). The H atoms of the water molecules were located from difference maps and refined isotropically.

### Crystal data

**Table:**

2C_3_H_7_N_6_^+^·C_6_H_2_N_2_O_4_^2^^−^·4H_2_O	*Z* = 2
*M**_r_* = 492.45	*F*(000) = 516
Triclinic, *P*1	*D*_x_ = 1.623 Mg m^−^^3^
Hall symbol: -P 1	Synchrotron radiation, λ = 0.73800 Å
*a* = 7.0200 (14) Å	Cell parameters from 130 reflections
*b* = 9.763 (2) Å	θ = 2.4–26.4°
*c* = 15.397 (3) Å	µ = 0.14 mm^−^^1^
α = 101.06 (3)°	*T* = 100 K
β = 99.82 (3)°	Needle, colourless
γ = 96.98 (3)°	0.15 × 0.04 × 0.03 mm
*V* = 1007.4 (4) Å^3^	

### Data collection

**Table:**

Huber single-axis diffractometer	4314 reflections with *I* > 2σ(*I*)
Radiation source: synchrotron, ESRF-CRG BM16	*R*_int_ = 0.086
Si 111	θ_max_ = 29.1°, θ_min_ = 3.5°
Detector resolution: 9.6 pixels mm^-1^	*h* = −9→9
CCD rotation images, thick slices scans	*k* = −12→12
34176 measured reflections	*l* = −20→20
4609 independent reflections	

### Refinement

**Table:**

Refinement on *F*^2^	Primary atom site location: structure-invariant direct methods
Least-squares matrix: full	Secondary atom site location: difference Fourier map
*R*[*F*^2^ > 2σ(*F*^2^)] = 0.043	Hydrogen site location: inferred from neighbouring sites
*wR*(*F*^2^) = 0.117	H atoms treated by a mixture of independent and constrained refinement
*S* = 1.07	*w* = 1/[σ^2^(*F*_o_^2^) + (0.0613*P*)^2^ + 0.6264*P*] where *P* = (*F*_o_^2^ + 2*F*_c_^2^)/3
4609 reflections	(Δ/σ)_max_ = 0.007
339 parameters	Δρ_max_ = 0.36 e Å^−^^3^
0 restraints	Δρ_min_ = −0.39 e Å^−^^3^

### Special details

**Table:**

Geometry. All esds (except the esd in the dihedral angle between two l.s. planes) are estimated using the full covariance matrix. The cell esds are taken into account individually in the estimation of esds in distances, angles and torsion angles; correlations between esds in cell parameters are only used when they are defined by crystal symmetry. An approximate (isotropic) treatment of cell esds is used for estimating esds involving l.s. planes.
Refinement. Refinement of *F*^2^ against ALL reflections. The weighted *R*-factor wR and goodness of fit *S* are based on *F*^2^, conventional *R*-factors *R* are based on *F*, with *F* set to zero for negative *F*^2^. The threshold expression of *F*^2^ > σ(*F*^2^) is used only for calculating *R*-factors(gt) etc. and is not relevant to the choice of reflections for refinement. *R*-factors based on *F*^2^ are statistically about twice as large as those based on *F*, and *R*- factors based on ALL data will be even larger.

### Fractional atomic coordinates and isotropic or equivalent isotropic displacement parameters (Å^2^)

**Table:**

	*x*	*y*	*z*	*U*_iso_*/*U*_eq_	
C1	0.08476 (18)	0.61767 (13)	0.86568 (7)	0.0064 (2)	
C2	0.16442 (17)	0.76548 (13)	0.92207 (8)	0.0062 (2)	
C3	0.32806 (19)	0.89895 (14)	1.05809 (8)	0.0108 (2)	
H3	0.3999	0.9071	1.1160	0.013*	
C4	0.28663 (19)	1.02060 (14)	1.02974 (8)	0.0104 (2)	
H4	0.3310	1.1079	1.0693	0.012*	
C5	0.12373 (17)	0.88757 (13)	0.89268 (8)	0.0071 (2)	
C6	0.00697 (19)	0.87874 (14)	0.79864 (8)	0.0090 (2)	
C7	0.14712 (17)	0.41260 (13)	0.60348 (8)	0.0060 (2)	
C8	0.26659 (17)	0.22077 (13)	0.65562 (8)	0.0062 (2)	
C9	0.16042 (17)	0.20889 (13)	0.50575 (8)	0.0062 (2)	
C10	−0.21253 (17)	0.27644 (13)	0.69392 (8)	0.0062 (2)	
C11	−0.32874 (17)	0.22388 (13)	0.54233 (8)	0.0065 (2)	
C12	−0.35228 (17)	0.44240 (13)	0.62008 (8)	0.0058 (2)	
N1	0.26800 (16)	0.77076 (12)	1.00488 (7)	0.0090 (2)	
N2	0.18487 (16)	1.01601 (11)	0.94733 (7)	0.0090 (2)	
N3	0.22544 (15)	0.35575 (11)	0.67361 (6)	0.0062 (2)	
H3A	0.2485	0.4042	0.7282	0.007*	
N4	0.23486 (15)	0.14484 (11)	0.57220 (7)	0.0065 (2)	
N5	0.11417 (15)	0.34110 (11)	0.51846 (7)	0.0066 (2)	
N6	0.10542 (16)	0.54193 (11)	0.62315 (7)	0.0083 (2)	
H6A	0.0564	0.5807	0.5806	0.010*	
H6B	0.1273	0.5874	0.6785	0.010*	
N7	0.34237 (16)	0.17081 (12)	0.72576 (7)	0.0097 (2)	
H7A	0.3722	0.0870	0.7176	0.012*	
H7B	0.3617	0.2222	0.7795	0.012*	
N8	0.13301 (16)	0.13572 (11)	0.42175 (7)	0.0093 (2)	
H8A	0.1619	0.0518	0.4112	0.011*	
H8B	0.0863	0.1720	0.3776	0.011*	
N9	−0.24368 (15)	0.18109 (11)	0.61670 (7)	0.0071 (2)	
N10	−0.38609 (15)	0.35169 (11)	0.54089 (7)	0.0065 (2)	
N11	−0.26557 (15)	0.40727 (11)	0.69764 (7)	0.0068 (2)	
H11	−0.2446	0.4672	0.7484	0.008*	
N12	−0.12818 (16)	0.24938 (11)	0.77090 (7)	0.0090 (2)	
H12A	−0.0920	0.1685	0.7718	0.011*	
H12B	−0.1094	0.3126	0.8201	0.011*	
N13	−0.35804 (16)	0.13295 (11)	0.46429 (7)	0.0098 (2)	
H13A	−0.3238	0.0509	0.4620	0.012*	
H13B	−0.4114	0.1557	0.4156	0.012*	
N14	−0.40337 (16)	0.56927 (11)	0.62636 (7)	0.0082 (2)	
H14A	−0.4592	0.5939	0.5788	0.010*	
H14B	−0.3808	0.6272	0.6780	0.010*	
O1	−0.08939 (13)	0.57080 (10)	0.86815 (6)	0.0115 (2)	
O2	0.19694 (13)	0.55284 (10)	0.82467 (6)	0.00925 (19)	
O3	0.00778 (18)	0.77035 (11)	0.74052 (6)	0.0206 (3)	
O4	−0.08449 (14)	0.97813 (10)	0.78707 (6)	0.0132 (2)	
O1W	0.56057 (15)	0.67742 (10)	0.81619 (6)	0.0126 (2)	
O2W	−0.48261 (15)	0.90431 (11)	0.72480 (6)	0.0137 (2)	
O3W	0.15750 (16)	1.31613 (11)	0.94758 (7)	0.0163 (2)	
O4W	0.49780 (15)	0.38403 (11)	0.88426 (6)	0.0142 (2)	
H1WA	0.454 (4)	0.635 (3)	0.8298 (16)	0.033 (6)*	
H1WB	0.665 (4)	0.644 (3)	0.8469 (18)	0.047 (7)*	
H2WA	−0.503 (4)	0.845 (3)	0.7578 (19)	0.051 (8)*	
H2WB	−0.348 (4)	0.933 (3)	0.7402 (19)	0.054 (8)*	
H3WA	0.151 (4)	1.213 (3)	0.9435 (17)	0.042 (6)*	
H3WB	0.144 (3)	1.353 (3)	1.0051 (17)	0.037 (6)*	
H4WA	0.390 (4)	0.379 (3)	0.9119 (18)	0.046 (7)*	
H4WB	0.589 (4)	0.362 (3)	0.9250 (18)	0.043 (7)*	

### Atomic displacement parameters (Å^2^)

**Table:**

	*U*^11^	*U*^22^	*U*^33^	*U*^12^	*U*^13^	*U*^23^
C1	0.0111 (5)	0.0052 (5)	0.0026 (5)	0.0015 (4)	−0.0003 (4)	0.0015 (4)
C2	0.0071 (5)	0.0074 (6)	0.0038 (5)	0.0015 (4)	0.0012 (4)	0.0002 (4)
C3	0.0126 (6)	0.0121 (6)	0.0051 (5)	0.0011 (5)	−0.0011 (4)	−0.0011 (4)
C4	0.0122 (5)	0.0086 (6)	0.0070 (5)	−0.0006 (5)	0.0002 (4)	−0.0036 (4)
C5	0.0086 (5)	0.0073 (6)	0.0051 (5)	0.0017 (4)	0.0016 (4)	−0.0001 (4)
C6	0.0142 (6)	0.0076 (6)	0.0055 (5)	0.0032 (5)	0.0008 (4)	0.0018 (4)
C7	0.0058 (5)	0.0065 (6)	0.0055 (5)	0.0009 (4)	0.0013 (4)	0.0012 (4)
C8	0.0062 (5)	0.0064 (6)	0.0063 (5)	0.0004 (4)	0.0018 (4)	0.0014 (4)
C9	0.0067 (5)	0.0057 (6)	0.0061 (5)	0.0007 (4)	0.0016 (4)	0.0014 (4)
C10	0.0061 (5)	0.0053 (6)	0.0073 (5)	−0.0001 (4)	0.0017 (4)	0.0022 (4)
C11	0.0065 (5)	0.0060 (6)	0.0067 (5)	0.0007 (4)	0.0009 (4)	0.0012 (4)
C12	0.0058 (5)	0.0053 (6)	0.0060 (5)	−0.0005 (4)	0.0012 (4)	0.0014 (4)
N1	0.0113 (5)	0.0098 (5)	0.0046 (4)	0.0020 (4)	−0.0007 (4)	0.0004 (4)
N2	0.0118 (5)	0.0066 (5)	0.0077 (5)	0.0009 (4)	0.0020 (4)	−0.0007 (4)
N3	0.0106 (5)	0.0044 (5)	0.0027 (4)	0.0013 (4)	0.0004 (3)	−0.0009 (3)
N4	0.0102 (5)	0.0047 (5)	0.0047 (4)	0.0015 (4)	0.0009 (4)	0.0012 (4)
N5	0.0102 (5)	0.0054 (5)	0.0043 (4)	0.0026 (4)	0.0013 (3)	0.0007 (4)
N6	0.0148 (5)	0.0060 (5)	0.0042 (4)	0.0048 (4)	0.0012 (4)	0.0001 (4)
N7	0.0171 (5)	0.0074 (5)	0.0045 (4)	0.0045 (4)	0.0000 (4)	0.0011 (4)
N8	0.0165 (5)	0.0070 (5)	0.0042 (5)	0.0052 (4)	−0.0001 (4)	0.0002 (4)
N9	0.0097 (5)	0.0060 (5)	0.0050 (5)	0.0021 (4)	−0.0001 (4)	0.0004 (4)
N10	0.0095 (5)	0.0047 (5)	0.0047 (4)	0.0015 (4)	0.0000 (3)	0.0008 (4)
N11	0.0112 (5)	0.0046 (5)	0.0034 (4)	0.0014 (4)	−0.0002 (4)	−0.0007 (3)
N12	0.0141 (5)	0.0067 (5)	0.0050 (5)	0.0025 (4)	−0.0011 (4)	0.0009 (4)
N13	0.0169 (5)	0.0068 (5)	0.0049 (5)	0.0055 (4)	−0.0013 (4)	−0.0003 (4)
N14	0.0139 (5)	0.0050 (5)	0.0050 (4)	0.0030 (4)	0.0001 (4)	−0.0001 (4)
O1	0.0109 (4)	0.0108 (5)	0.0097 (4)	−0.0016 (3)	0.0017 (3)	−0.0026 (3)
O2	0.0132 (4)	0.0073 (4)	0.0069 (4)	0.0035 (3)	0.0018 (3)	−0.0003 (3)
O3	0.0402 (6)	0.0143 (5)	0.0055 (4)	0.0160 (5)	−0.0039 (4)	−0.0020 (4)
O4	0.0189 (5)	0.0086 (5)	0.0116 (4)	0.0065 (4)	−0.0011 (4)	0.0023 (3)
O1W	0.0148 (5)	0.0107 (5)	0.0116 (4)	0.0004 (4)	0.0025 (3)	0.0020 (3)
O2W	0.0181 (5)	0.0127 (5)	0.0093 (4)	0.0024 (4)	−0.0007 (4)	0.0034 (4)
O3W	0.0225 (5)	0.0155 (5)	0.0119 (5)	0.0038 (4)	0.0052 (4)	0.0040 (4)
O4W	0.0160 (5)	0.0172 (5)	0.0102 (4)	0.0060 (4)	0.0013 (4)	0.0038 (4)

### Geometric parameters (Å, °)

**Table:**

C1—O2	1.2477 (15)	C11—N9	1.3613 (15)
C1—O1	1.2618 (16)	C11—N10	1.3599 (15)
C1—C2	1.5187 (17)	C12—N14	1.3214 (16)
C2—N1	1.3441 (15)	C12—N10	1.3278 (15)
C2—C5	1.3975 (17)	C12—N11	1.3691 (15)
C3—N1	1.3325 (17)	N3—H3A	0.8600
C3—C4	1.3876 (19)	N6—H6A	0.8600
C3—H3	0.9300	N6—H6B	0.8600
C4—N2	1.3354 (16)	N7—H7A	0.8600
C4—H4	0.9300	N7—H7B	0.8600
C5—N2	1.3438 (16)	N8—H8A	0.8600
C5—C6	1.5186 (17)	N8—H8B	0.8600
C6—O3	1.2491 (16)	N11—H11	0.8600
C6—O4	1.2507 (16)	N12—H12A	0.8600
C7—N6	1.3210 (16)	N12—H12B	0.8600
C7—N5	1.3271 (15)	N13—H13A	0.8600
C7—N3	1.3703 (15)	N13—H13B	0.8600
C8—N7	1.3253 (16)	N14—H14A	0.8600
C8—N4	1.3213 (16)	N14—H14B	0.8600
C8—N3	1.3699 (16)	O1W—H1WA	0.89 (3)
C9—N8	1.3217 (16)	O1W—H1WB	0.93 (3)
C9—N4	1.3615 (15)	O2W—H2WA	0.85 (3)
C9—N5	1.3551 (16)	O2W—H2WB	0.93 (3)
C10—N12	1.3205 (16)	O3W—H3WA	0.99 (3)
C10—N9	1.3286 (16)	O3W—H3WB	0.92 (3)
C10—N11	1.3669 (15)	O4W—H4WA	0.93 (3)
C11—N13	1.3176 (16)	O4W—H4WB	0.89 (3)
			
O2—C1—O1	126.43 (11)	N10—C12—N11	121.27 (11)
O2—C1—C2	118.10 (11)	C3—N1—C2	116.34 (11)
O1—C1—C2	115.41 (11)	C4—N2—C5	116.88 (11)
N1—C2—C5	122.02 (11)	C7—N3—C8	119.18 (10)
N1—C2—C1	115.11 (11)	C7—N3—H3A	120.4
C5—C2—C1	122.77 (10)	C8—N3—H3A	120.4
N1—C3—C4	122.00 (11)	C8—N4—C9	116.13 (11)
N1—C3—H3	119.0	C7—N5—C9	115.98 (11)
C4—C3—H3	119.0	C7—N6—H6A	120.0
N2—C4—C3	121.90 (12)	C7—N6—H6B	120.0
N2—C4—H4	119.0	H6A—N6—H6B	120.0
C3—C4—H4	119.0	C8—N7—H7A	120.0
N2—C5—C2	120.86 (11)	C8—N7—H7B	120.0
N2—C5—C6	118.16 (11)	H7A—N7—H7B	120.0
C2—C5—C6	120.98 (11)	C9—N8—H8A	120.0
O3—C6—O4	126.16 (12)	C9—N8—H8B	120.0
O3—C6—C5	116.43 (11)	H8A—N8—H8B	120.0
O4—C6—C5	117.39 (11)	C10—N9—C11	115.33 (11)
N6—C7—N5	120.70 (11)	C12—N10—C11	115.81 (10)
N6—C7—N3	117.75 (11)	C12—N11—C10	119.65 (10)
N5—C7—N3	121.55 (11)	C12—N11—H11	120.2
N7—C8—N4	121.63 (11)	C10—N11—H11	120.2
N7—C8—N3	116.81 (11)	C10—N12—H12A	120.0
N4—C8—N3	121.55 (11)	C10—N12—H12B	120.0
N8—C9—N4	116.83 (11)	H12A—N12—H12B	120.0
N8—C9—N5	117.57 (11)	C11—N13—H13A	120.0
N4—C9—N5	125.60 (11)	C11—N13—H13B	120.0
N12—C10—N9	121.52 (11)	H13A—N13—H13B	120.0
N12—C10—N11	116.74 (11)	C12—N14—H14A	120.0
N9—C10—N11	121.74 (11)	C12—N14—H14B	120.0
N13—C11—N9	116.94 (11)	H14A—N14—H14B	120.0
N13—C11—N10	116.87 (11)	H1WA—O1W—H1WB	105 (2)
N9—C11—N10	126.20 (11)	H2WA—O2W—H2WB	103 (2)
N14—C12—N10	120.75 (11)	H3WA—O3W—H3WB	105 (2)
N14—C12—N11	117.99 (11)	H4WA—O4W—H4WB	101 (2)

### Hydrogen-bond geometry (Å, °)

**Table:**

*D*—H···*A*	*D*—H	H···*A*	*D*···*A*	*D*—H···*A*
N3—H3A···O2	0.86	1.98	2.7726 (16)	153
N6—H6A···N5^i^	0.86	2.10	2.9449 (17)	169
N6—H6B···O3	0.86	2.19	2.8206 (17)	130
N6—H6B···O2	0.86	2.32	3.0378 (15)	141
N7—H7A···O2W^ii^	0.86	2.17	3.0080 (16)	164
N7—H7B···O4W	0.86	2.04	2.8407 (19)	156
N8—H8A···N9^iii^	0.86	2.39	3.2495 (17)	180
N8—H8B···O3^i^	0.86	2.03	2.8842 (16)	170
N11—H11···O1	0.86	1.98	2.7883 (17)	155
N12—H12A···O4^iv^	0.86	1.92	2.7557 (15)	162
N12—H12B···O3W^iv^	0.86	2.46	2.9981 (18)	121
N12—H12B···O1	0.86	2.47	3.1643 (17)	139
N13—H13A···N4^iii^	0.86	2.08	2.9275 (16)	167
N13—H13B···O2W^v^	0.86	2.10	2.8713 (16)	148
N14—H14B···O1W^vi^	0.86	2.21	2.9756 (16)	148
O1W—H1WA···O2	0.89 (3)	1.87 (2)	2.7263 (16)	163 (2)
O1W—H1WB···O1^vii^	0.93 (3)	1.95 (3)	2.8342 (16)	159 (2)
O2W—H2WA···O1W^vi^	0.85 (3)	2.07 (3)	2.8623 (16)	154 (3)
O2W—H2WB···O4	0.93 (3)	1.83 (3)	2.7533 (17)	171 (3)
O3W—H3WA···N2	0.99 (3)	1.97 (3)	2.9589 (16)	170 (2)
O3W—H3WB···O1^viii^	0.92 (3)	2.07 (3)	2.9810 (15)	176 (2)
O4W—H4WA···O3W^iv^	0.93 (3)	1.88 (3)	2.7846 (17)	164 (2)
O4W—H4WB···N1^ix^	0.89 (3)	2.08 (3)	2.9219 (17)	156 (2)

Symmetry codes: (i) −*x*, −*y*+1, −*z*+1; (ii) *x*+1, *y*−1, *z*; (iii) −*x*, −*y*, −*z*+1; (iv) *x*, *y*−1, *z*; (v) −*x*−1, −*y*+1, −*z*+1; (vi) *x*−1, *y*, *z*; (vii) *x*+1, *y*, *z*; (viii) −*x*, −*y*+2, −*z*+2; (ix) −*x*+1, −*y*+1, −*z*+2.
